# Supplementary material for: Are carbon emissions trading and green financial instruments synergistic? -Comprehensive quantitative research based on content analysis
Source: PLoS One. 2024 Mar 7;19(3):e0298601. doi: 10.1371/journal.pone.0298601 (PMC10919601; doi:10.1371/journal.pone.0298601)
Supplement: S1 Data — (ZIP) [file pone.0298601.s001.zip › Data/地级市Z_F工作报告.zip!/┴╔─■/╙¬┐┌╩╨/└┤╫╘╣1⁄2╓┌║┼í╛┬φ┐╦╩2╛▌═°í┐_2019.docx]

各位代表：

现在，我代表市政府向大会报告工作，请予审议，并请市政协委员提出意见。

一、2018年工作回顾

一年来，我们坚持以习近平新时代中国特色社会主义思想为指导，深入贯彻党的十九大精神，全面落实习近平总书记在辽宁考察时和在深入推进东北振兴座谈会上的重要讲话精神，在省委、省政府和市委的正确领导下，在市人大、市政协和全市人民的监督支持下，紧紧围绕“稳增长、促改革、调结构、惠民生、防风险、保稳定”各项任务，扎实开展“重强抓”专项行动，全市经济社会保持了平稳健康发展态势。

预计2018年，地区生产总值完成1350亿元，增长6%；固定资产投资435亿元，下降5.5%；一般公共预算收入131.3亿元，增长15.5%；社会消费品零售总额584亿元，增长10%；城镇常住居民人均可支配收入37000元，增长7.5%；农村常住居民人均可支配收入16826元，增长7.9%。

（一）强力推动“四基地一中心”建设，高质量发展迈出新步伐。

工业经济加速转型。围绕“四基地”发展定位，加快传统产业转型升级，石钢京诚新品开发、金辰机械太阳能组件等技术创新重点项目顺利实施，东盛实业智能工厂等一批智能制造示范项目建成投产。全年铝制品、钢镁深加工、石化、粮油加工和食品饮品产业产值分别达到137亿元、850亿元、245亿元和170亿元。规模以上工业增加值增长10.5%。

服务业稳步发展。着力建设物流与旅游产业中心，我市入选全国供应链创新与应用试点城市和港口型国家物流枢纽承载城市，营口传化公路港等一批项目投入运营，新增物流主体2474户。虹溪谷欢乐大世界等一批旅游项目建成运营，“鲅小鱼”智慧旅游服务平台正式开通，与传奇集团合作提升辽河老街文化旅游品牌，全年旅游总收入263.4亿元，增长12.1%。电子商务蓬勃发展，新增电商企业100余户。金融支持实体经济发展能力显著增强，“金小二”综合金融服务平台上线运行，新增金融机构5家，进入上市辅导期企业2户、辽股交挂牌企业20户、新三板挂牌企业1户、通过并购进入资本市场企业1户。全年服务业增加值增长5.5%。

乡村振兴开局良好。坚持以产业振兴为先导，加快转变农业发展方式，创建精品高效农业标准示范园6个，新建设施农业4310亩，新增农民专业合作社181个、家庭农场33个，滢辉海蜇、郑友和大酱等10个产品获评“2018辽宁礼物”。选派721名优秀干部服务乡村振兴，培育村级集体经济试点项目151个，农村发展后劲不断增强。熊岳镇入选全国综合实力千强镇、全国最美特色小镇50强，芦屯镇被列为国家农业产业强镇示范镇，高坎镇党家村被认定为国家级“一村一品”示范村。

创新驱动持续强化。全社会研发经费投入17.6亿元，增长18.5%。产学研合作不断深化，400余家企业与130余所院校、科研院所建立长期合作关系。知识产权试点城市建设扎实推进，每万人有效发明专利拥有量增长11.3%。加快创新创业平台发展，建设省级众创空间4个。新增高新技术企业40余家，高新技术产品产值实现560亿元，增长27.7%。

（二）突出抓好招商引资和项目建设，经济发展增添新动能。

招商引资成果丰硕。充分利用全国民营企业合作大会、营商大会等会展平台，大力实施产业链招商、产业集群招商、以商招商，全年实际利用外资1.46亿美元，引进国内实际到位资金278.5亿元，开工亿元以上项目252个，其中超50亿元项目8个。三峡集团燃机热电联产、中交营口LNG接收站等一批项目签约落地，忠旺铝产业园、中海石化DCC等项目加快建设。

对外交流合作深度拓展。出台对标先进工作实施方案，深化与先进地区交流合作。加快与盘锦协同发展，7项先期合作事项全面落实。国际交流日益深化，我市成为中国第二个“大图们倡议”物流委员会会员城市。

闲置资产有效盘活。积极推动闲置资产与企业、项目对接，开展二次招商、嫁接重组，中科北方成功收购北钢管业，新洲印刷投产运营。全年共清理盘活闲置土地595.4万平方米、闲置厂房48.2万平方米、闲置楼宇2.5万平方米，处置烂尾楼69.7万平方米。

（三）持续推进供给侧结构性改革，体制机制焕发新活力。

供给结构逐步改善。全面落实“三去一降一补”任务，大力淘汰落后产能，依法依规关停企业138家、整治453家；规模以上工业企业资产负债率同比回落2.1个百分点；销售商品房228万平方米，增长10%，库存进一步降低；全年为企业减负20.6亿元，为291户企业降低用电成本7亿元；全年民生投入161.3亿元，占财政支出的71%。

重点改革实现突破。国资国企改革不断深化，积极参与港口整合工作，组建城市基础设施建设投资集团、文化旅游集团，整合重组交运集团与物流集团，对燃气集团实行股权多元化改革。妥善解决国企改革历史遗留问题，全面完成“三供一业”分离移交任务，基本完成厂办大集体改革工作。政府机构改革顺利实施，设置组成部门32个，机构职能配置进一步优化；公益性事业单位改革初见成效，行政类事业单位改革成为全国样板，组建专门机构推动“四基地一中心”建设。深化园区改革创新，园区数量由26个减少至14个，全面推行公司化运作和全员竞聘管理。“飞地经济”加快发展，投产和在建项目19个，实际到位资金11.9亿元。扎实推进涉农资金整合，乡镇财政管理体制改革成效初步显现，乡镇一般公共预算收入增长16.4%。

民营经济快速发展。加强民营经济发展改革示范市建设，深入实施“个转企”“小升规”工程，全年完成“个转企”724户，新增规模以上工业企业79户、销售收入超百亿元企业3户，民间投资占固定资产投资比重达到84%，民营经济增加值占地区生产总值比重达到79.1%。辽宁望儿山酒业等3家企业获评“辽宁老字号”。国家发改委、全国工商联评估显示，营口是东北地区民营化程度最高、最具活力的城市。

营商环境持续优化。扎实推进“放管服”改革，向省级以上园区下放行政职权55项，建设项目在园区内实现闭环审批。辽河经济开发区区域评估试点有序推进，水保、地质灾害等5项评估事项实现一次编制、区域共享。事中事后监管进一步完善，行政执法检查事项“双随机、一公开”实现全覆盖。政务服务事项“网上办”比例达到82%，市本级政务服务中心即将运行。公共信用信息平台投入使用，社会信用体系建设不断加强。深入推进第三方评估建议整改，补齐营商环境短板，辽宁自贸区营口片区（以下简称“自贸区”）“以制度创新持续优化营商环境”经验做法得到国务院办公厅通报表扬。落实“项目管家”制度，对全市1549个项目（企业）实施“四个一”全周期帮扶，一批项目实现当年签约、当年落地、当年进资。“8890”政务便民服务平台整合运行，受理诉求电话4.7万件次，办复率100%。

（四）全面强化平台载体建设，对外开放形成新格局。

自贸区建设亮点纷呈。探索形成269项创新经验、63项典型案例，其中24项在全省复制，1项拟在全国推广；一般类工业项目取得施工许可证时间由120天压缩到15天，成为全省的标准蓝本。综合保税区（以下简称“综保区”）实现当年建设、当年验收，中央商务区投入使用，自贸区疏港铁路项目前期工作有序开展。规划年运量2000万吨的“中俄粮食走廊”项目签约落户，贸能港（营口）国际商品贸易中心启动运营。新增注册企业3151户，综合运营率达到35%，财政收入实现翻一番。

开放通道拓宽顺畅。积极推进营口港多式联运示范工程，中俄国际集装箱物流园区建设进展顺利，中欧班列辐射范围进一步扩大，“营海欧”通道第二条北极航线试航成功，全年海铁联运量达到82.3万标准箱，增长14%。营口港口岸开放取得阶段性成果，仙人岛港区通过国家验收。营口兰旗机场新增航线5条，全年完成旅客吞吐量近30万人次。

外经外贸稳定增长。加快推进与“一带一路”沿线国家的交流合作，启动辽宁“16+1”经贸合作示范区先行区建设。企业“走出去”步伐加快，金辰机械加纳光伏组件智能制造等一批项目有序推进。组织参加首届中国国际进口博览会，签约成交额超40亿元。积极扩大出口，全年出口总额完成246亿元，增长14%，增幅创6年来新高，占全省比重提升0.8个百分点。

（五）着力加强城市建设管理，城乡面貌实现新改善。

城乡规划管理不断加强。加快推进“多规合一”工作，编制完成营口市发展战略规划。深化城市管理综合执法改革，网格化管理实现城区全覆盖。积极推进智慧城市建设，“掌上新营口”上线运行，综合防控指挥系统等平台投入使用。

基础设施持续完善。扎实推进城乡基础设施扩容提质，实施立交桥维修改造工程，完成青花大街、盼盼路、聪慧路等道路新建及改造提升，交通拥堵压力进一步缓解。有序推进路灯、老旧小区等设施改造。加强“四好农村路”建设，全年修建农村公路392.4公里。投资1.83亿元实施新一轮农村电网改造升级工程，212个行政村、1.4万农户受益。

城市文化品质逐步提升。扎实开展“营口有礼”主题活动，一批影响城市文明、群众关心关注的突出问题得到有效解决，市民文明素养不断提高，文明有礼的社会风尚日益浓厚。百年气象站、沿海海防等系列文博馆建设加快推进，城市历史文化底蕴日益彰显。

（六）加快补齐短板弱项，三大攻坚战取得新进展。

防范化解重大风险成效明显。积极稳妥化解存量债务，政府债务平均利率由7.3%下降至4%。多渠道筹措资金96.3亿元，确保34.2万名企业离退休人员养老金及时足额发放。加大非法集资及互联网金融风险排查整治力度，高度关注并及时妥善处置企业财务风险，全市金融运行健康平稳。

精准脱贫扎实推进。大力实施“五个一批”工程，140家机关事业单位和43家企业定点帮扶。全年累计投入资金3.4亿元，实施产业项目252个，资助贫困学生1042人次，改造农村危房1088户，7315名贫困人员稳定脱贫，贫困村全部退出，扶贫工作取得阶段性成果。

污染防治有力有效。扎实推进中央环保督察及“回头看”、国家海洋督察反馈意见整改落实，永远角存量垃圾、大石桥镁产业治理加快推进，北部污水处理厂土建工程基本完工。取缔燃煤小锅炉811台，淘汰黄标车及老旧车辆11392台，PM2.5浓度下降6.9%。全面推行河长制，营柳河等黑臭水体治理任务如期完成。实施农村人居环境整治三年行动，集中清理农村垃圾，扎实推进“大棚房”专项整治，改造建设无害化厕所4223座，畜禽规模养殖场粪污处理设施装备配套率达到86%。

（七）坚持办实事解难事惠民生，百姓福祉得到新提升。

民生保障更加充分。21件重点民生实事按计划完成。城镇新增就业20852人，登记失业率2.47%。最低工资标准提高7.58%，企业退休人员养老金水平平均提高5.35%，城乡低保标准平均提高7.4%、9.4%，部分优抚对象等人员抚恤和生活补助标准以及特困人员救助供养标准平均提高10%。城镇居民医保和新农合年人均财政补助标准由450元提高到490元，基本公共卫生服务经费年人均财政补助标准由50元提高到55元。集中解决不动产登记历史遗留问题，109个小区、8.6万余户居民房屋完成不动产登记；安置超期回迁居民3942户；解决65户企业土地使用证办理难问题。

社会事业持续进步。教育教学质量不断提升，普惠性幼儿园学位覆盖率由42%提高到72%，小学“集团化”“小循环”办学模式试点初见成效，15名中小学教师获国家级教学竞赛一等奖。中小学素质教育基地基本建成，营口职业技术学院新校区投入使用，营口理工学院服务地方发展能力不断加强。医药卫生体制改革持续深化，分级诊疗制度初步建立，城市、县域医联体和专科联盟逐步推开。全科医师培训基地正式启用，第四人民医院晋升三级专科医院，引入社会资本建设的营口人民医院正式开诊，精准医学检验中心项目成功落地。加强文化体育建设，辽剧《龙凤镜》《三战张月娥》参加全国首届戏曲百戏盛典，组织“五进七送”活动300余场，完成100个农村文化（体育）广场建设改造，首届全民健身运动会成功举办。

稳定和谐局面巩固发展。深入开展“七五”普法，扎实推进平安营口建设，加快实施“雪亮工程”，扫黑除恶专项斗争取得阶段性成效，严重暴力犯罪案件下降17%，街面“两抢”案件下降53%。安全生产形势总体稳定，事故起数和死亡人数下降幅度均超过40%。积极化解信访积案，群众合理诉求得到妥善解决。有效处置非洲猪瘟疫情。

双拥共建、国防教育、海防海权、民兵预备役和人民防空工作深入开展；国家安全、民族宗教、外事侨务、食品药品安全、统计、史志、档案、气象、地震等工作不断加强；慈善、红十字、老龄、妇女儿童、残疾人等各项事业取得新成绩。

（八）深入实施“重强抓”专项行动，政府作风实现新转变。

市政府始终坚持正确的政治方向，在思想上政治上行动上同以习近平同志为核心的党中央保持高度一致。认真落实中央八项规定精神及其实施细则，坚决纠正和整治“四风”，全年“三公”经费下降11.4%。扎实开展“解放思想推动高质量发展大讨论”，强力推进“重强抓”专项行动，政府系统思想观念加快更新、工作作风持续转变、履职能力不断提升。严格依法行政，自觉接受市人大及其常委会法律监督和工作监督、市政协民主监督、社会舆论监督，建立人大代表、政协委员列席市政府常务会议制度，按期办复人大代表建议297件、政协提案278件、“直通车”建议28件、政协社情民意信息30件，办理质量持续提高。

各位代表：过去一年所取得的成绩来之不易，这是习近平新时代中国特色社会主义思想领航定向的结果，是省委、省政府和市委正确领导的结果，是市人大、市政协和社会各界监督支持的结果，是全市人民戮力同心、砥砺奋进的结果。在此，我代表市政府向全市人民，向各位投资者和企业家，向各位人大代表、政协委员、各民主党派、工商联、无党派人士和各人民团体，向离退休老领导、老同志，向驻营部队、武警官兵和中省直单位，向所有关心、支持营口建设的同志们、朋友们表示衷心的感谢并致以崇高的敬意！

各位代表：在总结成绩的同时，我们也清醒地看到，全市经济和社会发展仍然存在一些亟待解决的问题：一是受国际国内大环境影响，地区生产总值、固定资产投资增长率低于预期；二是产业转型升级进展较慢，创新能力不强，对外开放不够充分，区域发展不够均衡，发展质量和效益有待提高；三是民生领域还存在不少短板，优质教育、医疗等公共服务供给不足，城乡垃圾处理、城市供暖等历史欠账较多，城市品质还需提升；四是营商环境仍需持续改善，少数工作人员精神不振奋、作风不踏实，服务意识、服务能力、服务水平与先进地区仍存在较大差距，一些领域不正之风和腐败问题依然存在。这些问题是“四个短板”在我市的具体体现，根源在于思想不够解放、体制机制不够灵活。对此，我们一定要对标先进，解放思想，改革创新，破解难题，努力让全市人民看到真真切切的变化，获得实实在在的福祉。

二、2019年工作安排

今年是新中国成立70周年，是决胜全面建成小康社会第一个百年奋斗目标的关键之年，做好各项工作意义重大。当前，国内外形势错综复杂，面临的风险和困难明显增多。但要看到，我国发展仍处于并将长期处于重要战略机遇期，拥有足够的韧性、巨大的潜力，经济长期向好的态势不会改变。习近平总书记考察东北、辽宁期间发表的一系列重要讲话以及中央经济工作会议作出的战略部署，更为我们指明了前进方向、提供了根本遵循。特别是经过多年的发展，营口区位交通优势更加突出、开放平台更加完善、产业体系更加健全、民营经济更加活跃、营商环境更加优化；全市干部群众状态好、干劲足、信心满。只要我们在市委的正确领导下，审时度势、顺势而为，抓住有利条件，用好中央政策，就一定能够保持经济社会平稳健康发展。

今年政府工作的总体要求是：以习近平新时代中国特色社会主义思想为指导，认真落实习近平总书记在辽宁考察时和在深入推进东北振兴座谈会上的重要讲话精神、中央经济工作会议作出的战略部署，全面贯彻省委、省政府以及市委的要求，坚持稳中求进工作总基调，坚持新发展理念，坚持推动高质量发展，坚持以供给侧结构性改革为主线，坚持深化市场化改革、扩大高水平开放，继续打好三大攻坚战，扎实做好“六稳”工作，全力推进“四基地一中心”建设，强化自贸区引领作用，突出项目建设，做大临港产业，做强民营经济，加快补齐“四个短板”，以新气象新担当新作为，奋力开创营口全面振兴、全方位振兴新局面。

今年全市经济社会发展的主要预期目标是：地区生产总值增长6.5%左右，固定资产投资增长8%，一般公共预算收入增长7%，社会消费品零售总额增长7%，城乡居民收入增长与经济增长同步，城镇登记失业率控制在3%以内。

重点抓好以下六个方面工作：

（一）围绕优化营商环境、创新体制机制，全面深化各项改革。

以供给侧结构性改革为主线，坚持市场化取向和问题导向，突出关键领域，加大改革力度，激发新活力。

继续推动行政体制改革。全面完成机构改革，进一步调整理顺事业单位管理体制和运行机制，提高效率效能。整合组建市场监管、生态环保、文化市场、交通运输、农业领域综合执法队伍，推进市县两级综合行政执法体制改革，切实解决多头执法、多层执法和重复执法等突出问题。

持续深化“放管服”改革。以企业、群众需求为导向，继续梳理行政许可事项，能放尽放、能简尽简、能公开尽公开，构建阳光审批新机制。围绕“一网一门一次”目标，持续优化政务服务，市县两级服务事项网上可办率达到100%，与企业群众办事相关的服务事项全部实现“一门”办理并逐步向基层延伸，90%以上服务事项实现“最多跑一次”，工程建设项目审批时间压缩到80个工作日以内。全面实施“证照分离”改革，切实解决准入不准营的问题。推行“双随机、一公开”监管、信用监管、大数据监管、集成化综合监管、“告知承诺+事中事后监管”，持续提高监管效能。

统筹推进关键领域改革。分类分层实施国资国企改革，加快市属企业整合重组和混合所有制改革，有序实施热电、水务集团股权多元化改革。深化企业劳动人事分配三项制度改革，加快完善现代企业制度。健全国有资产监管方式，开展国有资本投资公司试点。加快财税体制改革，完善市与县（市）区财政管理体制，实行税收属地征管。严格落实化解政府债务三年行动工作方案，有效防控债务风险。推动金融创新，探索建立风险分担的信用贷款担保机制，设立金融创新奖励基金，引导金融机构开展产品与服务创新。实施土地出让收益分配改革，落实工业用地弹性出让、先租后让等制度。深化农村土地制度改革，基本完成农村土地确权登记颁证，成立营口市农村产权流转交易中心，推动农村土地流转。

做强做大民营经济。继续深化民营经济发展改革示范试点工作，制定并落实相关政策措施。加快股权融资平台、企业征信平台、融资担保平台、金融风险监测预警平台建设，推广无还本续贷模式，降低企业融资成本，全年新增金融机构3家，新增本外币贷款220亿元。支持企业股改上市，20户企业在辽股交挂牌。继续扩大电力直接交易规模，参与直购电企业达到330户。从劳动力供需两侧用力，努力缓解企业招工难问题。畅通政企对话交流渠道，健全企业家参与涉企政策制定机制，维护企业家合法权益。创造公平竞争环境，民企、国企、外企一视同仁。鼓励民营企业转型升级，实施“个转企”“小升规”“规升巨”工程，全年分别完成650户、50户和5户。民营经济增加值占地区生产总值比重突破80%。

全力优化营商环境。加强“诚信政府”建设，开展政府失信行为专项整治，着力解决说话不算、欠账不还、“新官不理旧账”等问题。构建信用联合奖惩机制，争创国家社会信用体系建设示范市，力争综合信用指数排名进入全国前50名。当好“项目管家”，深入落实项目（企业）“四个一”全周期帮扶制度，强化监督考评，真正实现精准有效帮扶。着力构建既“亲”又“清”的新型政商关系，多想“怎么办”、少说“不能办”，做到能办事、快办事、办好事，杜绝托人办事和“管卡压”“推绕拖”。

（二）围绕建设“四基地一中心”、加速新旧动能转换，大力实施创新驱动战略。

坚持以创新引领产业转型升级，推动新技术、新业态、新模式加快成长，努力实现高质量发展。

全面加强科技创新。围绕产业链部署创新链，以骨干企业为主体，组织开展一批重大产品研制和关键技术攻关，打造全链条、贯通式产业技术创新支撑体系。全社会研发经费投入增长15%。加快建设科技大市场和公共研发技术检验检测平台，完善科技成果转化机制，推动落地转化60项以上，技术合同成交额增长10%以上。坚持高新区、自贸区、综保区“三区”融合发展，加快集聚创新创业要素，推动高新区转型升级。加大高端人才和创新团队引进力度，全年培育省、市研发机构10家以上，新增国家级高新技术企业30家以上，高新技术产品产值增长15%。继续实施“企业·博士双创计划”，大力推动创新创业。

加快升级传统产业。围绕构建“432”现代工业体系，实施高质量发展工业项目50个，规模以上工业增加值增长11%。突出龙头带动、链条集聚，加快“四基地”建设，铝制品、钢镁深加工、石化、粮油加工和食品饮品产业产值突破1800亿元。编制大数据发展行动计划，推动制造业高端化、智能化改造，促进制造业与现代服务业融合发展，先进装备制造业增加值增长13%。做好供应链创新与应用试点工作，促进镁制品、汽保、农产品和物流、金融等产业相融合，推动单一业态向多业态演进，实现转型升级、做大做强。加快国家农产品冷链流通标准化试点建设，发展无车承运人等新型运输方式，推进运输结构调整，提升物流业发展水平。大力实施“旅游+”战略，突出抓好大白鲸海岸城、辽河老街改造等项目建设，推进旅游业提质增效，全年物流和旅游产业增加值占服务业增加值比重突破55%。积极培育消费热点，满足多层次消费需求。多措并举去库存，促进房地产市场健康发展。

壮大发展新兴产业。积极引进培育新材料、新能源等战略性新兴产业，加快金能移动能源产业园等项目建设，全年战略性新兴产业产值增长15%。引导电商企业与快递物流园区合作，扩大云仓服务覆盖率，做大电子商务产业规模。大力培育海洋康养、温泉康养等新业态，建设北海康养示范基地。深入开展“文化+”行动，提升文化产业发展水平。继续办好鲅鱼圈国际马拉松等各类赛事节庆活动，积极引进国内外高端会议展览，繁荣会展经济。

（三）围绕激发区域潜力、优化资源配置，加快构建协调发展新格局。

突出项目驱动和园区支撑，扎实推进乡村振兴，促进城乡互动互补、融合发展，加快破除城乡二元结构。

更加聚焦招商引资项目建设。紧紧围绕主导产业，瞄准京津冀、长三角、珠三角和日韩俄、中东欧等地区和国家，依托中国国际进口博览会、夏季达沃斯论坛等平台以及“知名企业营口行”等招商活动，突出抓好产业链招商、以商招商、“飞地”招商，全年实际利用外资增长15%以上，引进国内实际到位资金增长10%以上。推进胜星石化二期、鑫钰汽车轮毂等一批项目开工建设。继续清理盘活闲置资产，实现“腾笼换鸟”，激发闲置资产活力。

深入推进园区创新发展。以产业园区为平台，按照产业定位，集中力量补齐延长产业链，做大做强主导产业。坚持以体制机制创新引领园区发展，持续深化“管委会+公司”、全员聘任制等改革，优化要素资源配置，打造加速发展“主引擎”。依托园区，大力发展“飞地经济”，破解乡镇项目落地瓶颈，力争年内所有乡镇都有“飞地”项目落地，形成园区承载、全域联动的发展格局。

大力实施乡村振兴战略。坚持把做大做强农村产业作为乡村振兴的首要任务，围绕发展精品农业、特色农业，深化农业供给侧结构性改革，合理调整“粮经饲”结构，新增设施农业5000亩。提升现代农业示范区和农产品加工集聚区建设水平，新建续建千万元以上农业产业化项目10个，农产品加工集聚区主营业务收入增长7%以上。继续办好海蜇节，建设海蜇出口加工区和全国海蜇集散地。大力发展休闲农业，促进农业与旅游观光、健康养生等多种业态相融合，探索发展田园综合体，推动乡村经济由卖产品向卖服务、卖体验转变。培育家庭农场、农民专业合作社等新型经营主体，完善治理机制，统筹整合涉农资金，壮大村集体经济。全面落实加快县域经济发展三年行动计划，增强县域综合实力。

（四）围绕解决环境突出问题、加强生态保护修复，巩固提升绿色发展优势。

牢固树立“绿水青山就是金山银山、冰天雪地也是金山银山”的理念，深入落实生态环境保护工作责任，全面提升生态文明建设水平。

着力解决突出环境问题。加大中央环保督察及“回头看”、国家海洋督察反馈意见整改力度，加快大石桥镁产业治理等重点问题整改。实施城乡垃圾综合整治工程，形成垃圾闭环处理体系，按期完成永远角存量垃圾治理。

切实抓好生态系统保护。坚持铁腕治霾，加快镁质材料、钢铁等重点行业专项整治，逐步淘汰城市建成区20蒸吨/小时及以下燃煤锅炉，空气质量优良天数比率达到73.4%，让群众感受到空气质量持续好转。把治水放到更加重要位置，严格落实河长制，加强水源地保护，完成民兴河等黑臭水体以及入海河流整治，高标准打好渤海治理攻坚战。开展土壤污染防治行动。继续实施生态绿化工程，全年义务植树100万株。深入开展农村环境综合整治，重点做好垃圾污水处理、农村卫生厕所新建改造、村容村貌提升，建设美丽乡村。

积极倡导绿色发展方式。提高项目准入门槛，坚决不新上高耗能、高污染、高排放等不符合国家产业政策的项目。深入推进“电化营口”，大力推广煤改电等电能替代项目，推动镁质材料、钢铁、石化等行业进行清洁生产与节能技术改造，逐步形成绿色生产方式，全年单位地区生产总值能耗下降3.4%。实施非煤矿山“五矿共治”，集中开展矿业权控增减存等专项行动，着力解决矿山“多、小、散”等突出问题。

（五）围绕实现港口自贸区联动发展、融入“一带一路”建设，努力打造开放合作高地。

坚持全球化视野、国际化思维，依托港口、自贸区、综保区等平台优势，着力提升对外开放能级水平。

大力发展临港经济。推进“营满欧”“营新欧”“营海欧”大通道及沿线物流节点建设，提高营口港中欧班列联运水平，加快形成多式联运、无缝对接的国际大通道。扎实推进港口型国家物流枢纽建设，编制物流产业发展规划，构建海港、空港、陆港“三港联动”的物流体系，推动港口功能向腹地延伸。建设粮食、原糖、镁制品等大宗商品国际贸易平台和现货交易市场，完善税收等相关政策，促进货值和税收落地，打造东北地区现代大宗商品物流中心。围绕构建全域临港产业区，推进已落地重大项目建设，加快形成以装备制造、石化等传统产业为基础，以新能源、新材料等新兴产业为支撑的临港工业体系。

充分发挥自贸区引领示范作用。立足首创性、差异化、可复制、可推广，围绕多式联运大通道建设以及自贸区、综保区和高新区“三区”叠加的能级释放开展集成创新，推出具有营口特色的全流程、全链条制度创新成果。向全市复制推广创新成果20项以上，充分发挥外溢效应。围绕综保区封关运行，大力引进保税加工、保税物流等项目，加快发展保税研发、金融结算等新型服务贸易，提升进出口贸易规模和水平。研究制定与国际接轨的税收服务举措，探索建设金融创新平台，打造航运金融、制造业金融、科技金融、跨境金融“四位一体”的金融服务综合体。推动自贸区疏港铁路建设，促进港口与自贸区联动发展。全年新增注册企业超2500户，一般公共预算收入增长50%。

加快推动对外贸易转型升级。围绕打造辽宁“16+1”经贸合作示范区先行区，筹备建设中东欧16国国别馆，与中东欧国家开展双向经贸合作。持续完善国际贸易“单一窗口”功能，进一步提升贸易便利化水平。多措并举稳定出口，开拓“一带一路”沿线国家新市场。鼓励生产型企业开展外贸业务，支持东盛实业打造外贸综合服务平台。加快培育贸易新业态新模式，重点推进“中俄粮食走廊”和贸能港（营口）国际商品贸易中心等项目，大力发展跨境电子商务，努力在营口实现“买全球、卖全球”。全年进出口占全省份额实现稳中有升。

持续扩大开放合作。依托俄罗斯特维尔市、韩国光阳市等国际友城，以经贸交流为重点，开展多领域务实合作。积极对接京津冀协同发展、长江经济带发展、粤港澳大湾区建设等国家重大战略，深化与连云港对口合作及友好城市交流合作，促进先进产能、技术落户。建设沈阳经济区出海大通道，抢抓大连太平湾临港经济区建设机遇，力争将鲅鱼圈区、仙人岛开发区和太平湾临港经济区统筹考虑，整体建设。加快营口、盘锦协同发展，推动合作事项落实落地。

（六）围绕破解民生难题、补齐民生短板，提高人民群众获得感幸福感安全感。

始终把群众的安危冷暖放在心上、抓在手中，在发展中保障和改善民生。今年，将集中力量精心办好15件民生实事，推动改革发展成果更多更公平惠及全市人民。

坚决打赢脱贫攻坚战。继续抓好以产业扶贫为重点的“五个一批”工程，充分调动帮扶单位和派驻干部积极性，全年实施特色产业增收项目30个，新增扶贫农民合作组织20个。妥善安排贫困户子女就业，有效解决好因病致贫、因贫失学问题。全年3500名贫困人口实现稳定脱贫。

强化就业和社会保障。把稳就业摆在突出位置，重点抓好弱势群体就业工作，全年新增就业1.8万人，离校未就业高校毕业生就业率80%以上。继续提高企业退休人员基本养老金、城乡居民医保财政补助、城乡低保等标准。加大保费征缴和财政补助力度，保证养老金及时足额发放。整合城乡居民医保制度，完善医保支付政策。全面实施特困人员救助供养制度，确保有需求的城乡特困人员集中供养全覆盖。继续解决不动产登记难、回迁难等历史遗留问题。

统筹社会事业发展。提升各级各类教育水平，加快学前教育普及普惠发展，分步实施中小学校提标改造，扎实推进新高考改革和高中办学质量提升，支持高、中等职业院校与企业实现产教融合、校企合作，做好营口理工学院本科教学以评促建工作。深化医药卫生体制改革，实施公共卫生服务和城区医疗技术服务能力提升工程，全面提高医疗水平。加强食品药品监管，提升食品药品检验能力，保障城乡居民饮食和用药安全。推进社区居家养老服务改革试点，完善农村养老机构和设施，吸引社会资本参与养老服务。深入实施文化惠民工程，开展下乡惠民演出100场，建设改造农村文化（体育）广场100个，完善公共文化场馆服务功能。加强文化遗产保护，继续推进系列文博馆建设，办好望儿山母爱文化节等系列活动，不断提升城市文化软实力。

加强城乡建设管理。坚持以战略规划为引领，编制空间规划，推动北部城区和大石桥、盖州和北海、鲅鱼圈和仙人岛一体化发展。实施路网提升工程，新建改造体育路、智胜街、智新街等道路。完成热源新建改造工程，新增供热能力300万平方米。加快智慧城市建设，完善信息基础设施，新建1000个5G基站。启动立体停车楼和新能源汽车充电桩建设。推动老旧小区改造，优化物业管理服务，加大市政基础设施维护管理力度，提升居住品质。完善沿海产业基地、营东新城生产生活服务设施，促进新老城区资源共享。完成“四好农村路”新改建30公里、维修改造120公里。实施机动车停车管理条例等法律法规，提升城市管理规范化水平。

推动“营口有礼”主题活动常态化长效化。坚持以创建全国文明城市和打造最优营商环境为主线，大力弘扬新时代“辽宁精神”，实施好“七个一百”文明创建工程，深化文明礼德进机关、进社区、进乡村等“十进”行动，全力打造“营口有礼”城市名片。

提升社会治理能力。持续抓好“七五”普法，提高法治营口建设水平。全面加强社会治安防控体系建设，深入开展扫黑除恶专项斗争，严厉打击各类违法犯罪活动，维护良好社会秩序。强化源头管控和纠纷调处，依法解决群众合理信访诉求，化解信访存量30%以上。做好安全生产和应急救援工作，加大安全隐患排查整治力度，坚决遏制重特大事故发生。

加强国防教育、民兵预备役建设，深化军民融合发展，积极开展双拥共建活动，持续推进“四城同创”；做好国家安全、民族宗教、外事、统计、物价监管、人民防空、海防海权、气象、地震等工作；加快发展慈善、红十字、老龄、妇女儿童、残疾人等各项事业。

三、以“重强抓”专项行动为平台，持续加强政府自身建设

坚持把党的领导贯穿于政府工作各方面各环节，深化“重强抓”专项行动，锲而不舍地抓好政府自身建设，不断提升施政能力和服务水平。

强化政治建设。始终把政治建设摆在首位，树牢“四个意识”、坚定“四个自信”、坚决做到“两个维护”，始终同以习近平同志为核心的党中央保持高度一致，更加坚决地贯彻中央决策部署，更加自觉地服从省委、省政府和市委领导，保持政治清醒。

提升施政本领。深入开展“解放思想推动高质量发展大讨论”，坚持用习近平新时代中国特色社会主义思想武装头脑、指导实践、推动工作。在全市政府系统营造自觉学习、扎实工作、勇于担当的浓厚氛围，着力提升科学发展本领、改革创新本领、狠抓落实本领。

严格依法行政。始终把法治思维、法治方式贯穿于决策、执行和监督全过程。认真落实市人大及其常委会作出的决议、决定，依法接受市人大的法律监督和工作监督，自觉接受市政协的民主监督，主动接受群众监督和社会舆论监督。继续探索人大代表、政协委员和人民群众参与政府工作的有效途径。全面推进政务公开，让权力在阳光下运行。

持续转变作风。牢固树立“店小二”意识，深入推进服务型政府建设。始终做到“实”字当头，讲实话、察实情、谋实招、办实事、求实效。大力推动政府工作系统化、目标化、标准化，做到目标明确、节点清楚、标准具体、责任到位，确保干一件成一件、干一桩成一桩。严格落实中央八项规定精神及其实施细则，坚决反对形式主义、官僚主义，重点解决“表态多调门高、行动少落实差”、重“痕”不重“绩”、留“迹”不留“心”以及“上热下冷”等问题。

保持廉洁本色。认真落实党风廉政建设主体责任和“一岗双责”制度，强化廉政警示教育，以更高标准抓好政府系统反腐倡廉工作。坚持用制度管人管权管事，紧盯工程建设、政府采购等重点领域和关键环节，加强日常监督和审计监督，扎紧织密制度“笼子”，铲除腐败滋生土壤，努力打造忠诚、干净、担当的公务人员队伍。

各位代表：大道至简，实干为要。实现全年既定目标任务不是等得来、喊得来的，而是拼出来、干出来的。让我们高举习近平新时代中国特色社会主义思想伟大旗帜，更加紧密地团结在以习近平同志为核心的党中央周围，在省委、省政府和市委正确领导下，解放思想、锐意进取，求真务实、埋头苦干，以优异成绩迎接中华人民共和国成立70周年！
